# Dynamics of Microbial Communities during the Removal of Copper and Zinc in a Sulfate-Reducing Bioreactor with a Limestone Pre-Column System

**DOI:** 10.3390/ijerph19031484

**Published:** 2022-01-28

**Authors:** Aracely Zambrano-Romero, Dario X. Ramirez-Villacis, Gabriel Trueba, Reyes Sierra-Alvarez, Antonio Leon-Reyes, Paul Cardenas, Valeria Ochoa-Herrera

**Affiliations:** 1 Instituto de Microbiología, Universidad San Francisco de Quito USFQ, Diego de Robles y Via Interoceánica, Quito 17-1200-841, Ecuador; mazambranor@usfq.edu.ec (A.Z.-R.); gtrueba@usfq.edu.ec (G.T.); pacardenas@usfq.edu.ec (P.C.); 2 Colegio de Ciencias e Ingeniería, Instituto Biósfera, Universidad San Francisco de Quito USFQ, Diego de Robles y Vía Interoceánica, Quito 17-1200-841, Ecuador; dxramirez@usfq.edu.ec (D.X.R.-V.); aleon@usfq.edu.ec (A.L.-R.); 3 Laboratorio de Biotecnología Agrícola y de Alimentos, Universidad San Francisco de Quito USFQ, Diego de Robles y Vía Interoceánica, Quito 17-1200-841, Ecuador; 4 Department of Chemical and Environmental Engineering, The University of Arizona, Tucson, AZ 85721, USA; rsierra@mail.arizona.edu; 5 Escuela de Ingeniería, Ciencia y Tecnología, Universidad del Rosario, Bogotá 111221, Colombia; 6 Department of Environmental Sciences and Engineering, Gillings School of Global Public Health, University of North Carolina at Chapel Hill, Chapel Hill, NC 2759, USA

**Keywords:** sulfate-reducing, bioreactor, anaerobic, acid rock drainage, metagenomics, microbial, community, dynamics, diversity

## Abstract

Biological treatment using sulfate-reducing bacteria (SRB) is a promising approach to remediate acid rock drainage (ARD). Our purpose was to assess the performance of a sequential system consisting of a limestone bed filter followed by a sulfate-reducing bioreactor treating synthetic ARD for 375 days and to evaluate changes in microbial composition. The treatment system was effective in increasing the pH of the ARD from 2.7 to 7.5 and removed total Cu(II) and Zn(II) concentrations by up to 99.8% and 99.9%, respectively. The presence of sulfate in ARD promoted sulfidogenesis and changed the diversity and structure of the microbial communities. *Methansarcina* spp. was the most abundant amplicon sequence variant (ASV); however, methane production was not detected. Biodiversity indexes decreased over time with the bioreactor operation, whereas SRB abundance remained stable. *Desulfobacteraceae*, *Desulfocurvus*, *Desulfobulbaceae* and *Desulfovibrio* became more abundant, while *Desulfuromonadales*, *Desulfotomaculum* and *Desulfobacca* decreased. *Geobacter* and *Syntrophobacter* were enriched with bioreactor operation time. At the beginning, ASVs with relative abundance <2% represented 65% of the microbial community and 21% at the end of the study period. Thus, the results show that the microbial community gradually lost diversity while the treatment system was highly efficient in remediating ARD.

## 1. Introduction

Massive generation of acid rock drainage (ARD) is one of the most important environmental impacts of mining activities. ARD contains high concentrations of heavy metals, metalloids and sulfate, and low pH values [1,2,3].

Bioremediation of ARD through metal bioprecipitation is mediated by sulfate reducing bacteria (SRB), a diverse group of microorganisms which use sulfate as a terminal electron acceptor [4,5,6,7,8]. In the presence of a suitable electron-donating substrate, either an organic compound or hydrogen, SBRs catalyze the reduction of sulfate into sulfide. Sulfide anions react with many metal cations (e.g., Cu(II), Zn(II), Ni(II), Hg(II), Cd(II), etc.) forming sparingly soluble metal sulfides that can be easily removed from solution by gravity settling sulfides [5,9,10]. In addition, the microbial oxidation of the electron-donating substrate by SRB generates alkalinity and increases pH [6,9,11,12].

SRBs constitute a heterogeneous bacterial group of ubiquitous, metabolically flexible, and free-living microorganisms [7,13]. Both in natural and in engineered environments, SRBs are part of consortia with other anaerobic microorganisms, such as methanogenic archaea and syntrophic bacteria [5,14,15,16]. The assessment of the synergistic or antagonistic effects between members of a microbial consortium and the multiple interactions influenced by contaminants, metabolites, and inhibitory substances, is crucial in the development and improvement of innovative bioremediation [17,18,19].

The effects of heavy metals, pH and different electron donors and carbon sources on sulfate reduction and methanogenesis in anaerobic bioreactors are well-documented [12,20,21,22,23,24]. However, less information is available on the temporal changes in the structure of the microbial communities that drive the sulfate reduction process in bioreactors treating ARD [15,25,26].

Microbial community dynamics are linked to the ecological functionality of community members and adaptation to external disturbances [24,27,28,29]. Therefore, we hypothesize that metal-bioprecipitation of the influent may influence the microbial communities and may result in the assembly of a robust and long-lasting consortium for ARD bioremediation.

The objective of this study was to evaluate the performance of a sequential system, consisting of a limestone bed filter followed by a sulfate-reducing bioreactor, treating synthetic ARD composed of copper (II), zinc (II), sulfate and acetate as an organic carbon source, and to characterize the diversity of the microbial communities responsible for the biological treatment of ARD during 375 days of operation. We used 16S ribosomal RNA (rRNA) gene sequencing to assess microbial community structure, abundance, and diversity.

## 2. Materials and Methods

### 2.1. Configuration and Operation of the Treatment System

**Figure 1:**
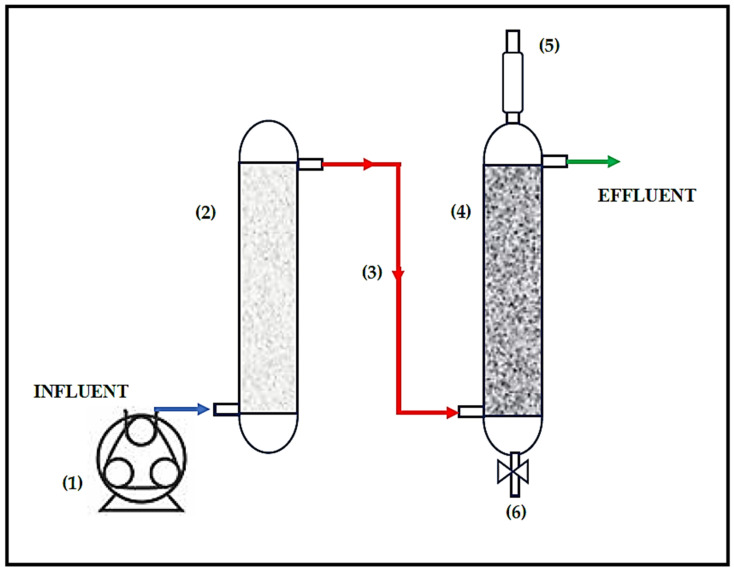
Scheme of the treatment system. (1) Peristaltic pump, (2) limestone pre-column (height: 25 cm, internal diameter (i.d.): 5.5 cm), (3) limestone pre-column effluent and/or bioreactor influent, (4) biological reactor (height: 43.2 cm, i.d.: 5.5 cm), (5) biogas, (6) sampling port of sludge.

The pilot-scale sulfate-reducing (SR) bioreactor with a limestone pre-column (Figure 1) was described in a previous study [30]. The volume of the limestone precolumn was 0.40-L and the volume of the biological reactor was 0.49-L. Before starting our research, the system pre-column was packed with fresh and pre-sieved limestone, as recommended by Mendez and coworkers [30].

The treatment system was fed with a synthetic ARD composed of a basal mineral medium [30] supplemented with sulfate (2000 mg L^−1^), acetate as an electron donor and an organic carbon source with a chemical oxygen demand (COD) of 2500 mg COD L^−1^, Cu(II) (15 mg L^−1^) and Zn(II) (15 mg L^−1^). The medium pH was adjusted to 2.7 by adding hydrochloric acid (HCl). The reactor was operated at 30 ± 2 °C under darkness conditions to avoid anoxygenic photosynthesis. The inoculum (sludge) contained 7.18% volatile suspended solids (VSS) in wet-weight, with a maximum sulfate reducing activity of 4.25 mg S^2−^ L^−1^ d^−1^.

During period I (day 0–24), the bioreactor was operated as a stand-alone reactor without metal addition or acidification at a volumetric loading rate of 2.8 g acetate-COD L^−1^ d^−1^ and a hydraulic retention time (HRT) of 2.0 ± 0.37 d. In periods II and III, the bioreactor was operated for 201 and 150 days, respectively, with the lime-stone bed reactor. Due to the toxicity of the acidic conditions and high metal concentrations of the influent, the purpose of the limestone reactor was to increase pH by alkalinity generation and to reduce heavy metal levels by precipitation as hydroxides or carbonates [11,31]. In period II, 15 mg L^−1^ of Cu(II) was added (as CuCl_2_·2H_2_O), and, in period III, in addition to Cu(II), 15 mg L^−1^ of Zn(II) was dosed (as ZnCl_2_). The volumetric loading rate in periods II and III averaged 2.8 g acetate-COD L^−1^ d^−1^ with an HRT of 2.0 ± 0.37 d. For preparation of synthetic ARD, concentrations of Cu(II) and Zn(II) were selected to simulate the metal concentrations found in real ARD collected in mining zones in Ecuador [30]. Influent and effluent samples were sampled and analyzed weekly. During period I, the pH of the influent was not modified, while in periods II and III, the influent pH was acidified to a value of 2.7.

### 2.2. Analytical Methods

Sulfate was determined using the turbidimetric method 4500-SO_4_^2−^ [32]. Sulfide was analyzed immediately after sample collection using the methylene blue method [33]. Chemical oxygen demand was determined by the colorimetric method 5220D according to standard methods [32]. Before sulfate, COD and metals analyses, samples were filtered using a 0.45-µm filter. Sulfate reduction and COD removal were determined as the difference between the influent and the effluent sulfate and COD concentrations, respectively.

pH was measured using a portable multi-parameter Thermo Scientific Orion 5-Star meter (Thermo Scientific, Beverly, MA, USA) according to the methods 2510A and 4500A, respectively [32].

Soluble metal concentrations of Cu(II) and Zn(II) in liquid samples were analyzed by inductively coupled plasma optical emission spectrometry (ICP-OES, Thermo Scientific ICAP 7400, Thermo Scientific, Beverly, MA, USA). Calibration curves were conducted for each metal in 2% HNO_3_ trace metals. Samples were constructed according to method 3120B [32].

Methane (CH_4_) produced in the sulfate-reducing bioreactor was estimated by the liquid displacement method through a sodium hydroxide (NaOH) solution (2%) to remove carbon dioxide (CO_2_) and hydrogen sulfide (H_2_S) [34]. The H_2_S concentration in the biogas was calculated from the H_2_S concentration in the liquid phase, assuming equilibrium between phases and a dimensionless Henry’s factor of 0.36 [20,35]. The electron equivalents of reducing power, as percentage, fed to the reactor (COD_in_, as g COD L^−1^ reactor d^−1^) used for methane (% CH_4_-COD) and sulfide (% H_2_S-COD) generation, were calculated as described in our previous publication [20].

Standard deviations and analysis of variance (ANOVA), were calculated using SPSS Statistics software (IBM Corp., New York, NY, USA). Thus, the media values of analytical parameters in the different operational periods were compared by ANOVA, with 95% confidence intervals.

### 2.3. Sludge Sample Collection

Samples were collected for 375 days in sterile plastic recipients. Two samples of sludge were collected during period I (adaptation phase), day 0 and day 24, two during period II (15 mg Cu(II) L^−1^), day 181 and day 217, and two during period III (15 mg Cu(II) L^−1^ and 15 mg Zn(II) L^−1^), day 356 and day 372, six-bioreactor sludge samples in total [35]. Approximately 15–20 g of material was collected per sample. Genomic DNA was immediately extracted and maintained at −80 °C prior to molecular analyses.

### 2.4. DNA Extraction

DNA extraction was performed as previously described (Ben-Dov et al., 2007), using DNeasy^®^ PowerSoil^®^ Kit (QIAGEN GmbH., Hilden, Germany). The concentration of the resulting DNA preparation was determined by a Qubit^®^ fluorometer (Thermo Fisher Scientific Inc, Waltham, USA). DNA integrity was evidenced by electrophoresis and DNA samples were stored at −80 °C until use in molecular analysis [35]. For 16S rRNA analysis, DNA samples were lyophilized in a freeze dryer (ilShin BioBase, Dongducheon, Korea), prior to submission for next generation sequencing.

### 2.5. rRNA Analysis

V3 and V4 regions of 16S rRNA genes were sequenced (Primers: Bakt_341F: CCTACGGGNGGCWGCAG and Bakt_805R: GACTACHVGGGTATCTAATCC) in Macrogen (Seoul, Korea) using an Illumina MiSeq platform (Albany, NY, USA) and MCS Sequencing Control Software, yielding a total of ~120.3 Mbp of sequencing reads. Only reads with 100% correct primer sequences were used. After primer sequences were removed, the resulting sequences were collapsed into amplicon sequence variants (ASVs) using the R package DADA2 version 1.8.1 [36,37]. A total of 766,178 sequences were merged in 703 ASVs. Taxonomic assignment of each ASV was performed using the naïve Bayes k-mer method implemented in the MOTHUR package [38] using the Silva 132 database as the training reference [39]. All samples were rarefied to 69,000 reads per sample before analysis.

### 2.6. Microbial Diversity and Statistical Analysis

Alpha diversity metrics and the Bray–Curtis dissimilarity index were calculated using the vegan package version 2.5–3 [40]. Five measures for the sludge diversity and richness were used: Shannon index (diversity index), inverse Simpson index (to quantify average proportional abundance of species in a sample), richness (count of unique species), Chao1 (abundance of species-based index), and evenness (how equally abundant species are) [41]. Beta-diversity analyses (principal coordinate analysis) were based on Bray–Curtis dissimilarity calculated from the rarefied abundance tables [42,43]. To visualize the composition of the communities, a stacked bar chart was built by grouping all ASVs assigned to known methanogenic archaea [44], non-syntrophic SRB [45] and syntrophic SRB [46]; then, all the remaining ASVs with an abundance higher than 2% were collapsed to genus level. A heatmap plotting log10 of the counts for methanogenic archaea, SRB and syntrophic bacteria ASVs was created using the Pheatmap package [47]. Finally, a phylogenetic tree was assembled using the ASV sequences of the methanogenic archaea, non-syntrophic SRB and syntrophic SRB using MEGA-X software [48]. The sequences were aligned using the default parameters of the Clustal-W algorithm [49,50] and a maximum likelihood tree was constructed using the GTR+G+I model with 500 bootstrap replications [48]. The final consensus tree (70% cut-off value) was visualized and annotated with the online tool ‘Interactive Tree of Life’ (https://itol.embl.de (accessed on 27 August 2021)) [51].

## 3. Results

### 3.1. Performance of the Treatment System

The sulfate-reducing bioreactor with a limestone pre-column (Figure 1) was operated for 375 days. In period I, or the phase of adaptation, the treatment system was fed with non-acidified synthetic ARD (pH: 8.04 ± 0.36). In period II, 15 mg L^−1^ of Cu(II) was added, and the pH of synthetic ARD was 2.7. In period III, 15 mg L^−1^ of Cu(II) and 15 mg L^−1^ of Zn(II) were simultaneously added to the pH-2.7 synthetic ARD (Table 1).

**Figure 2:**
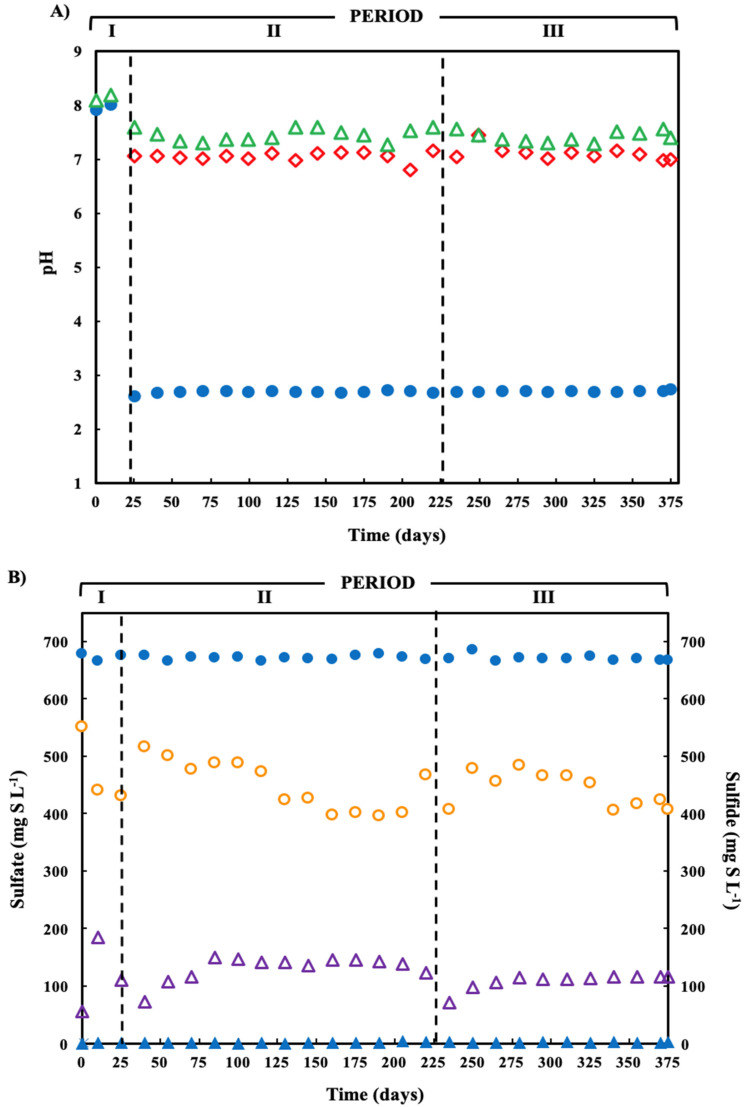

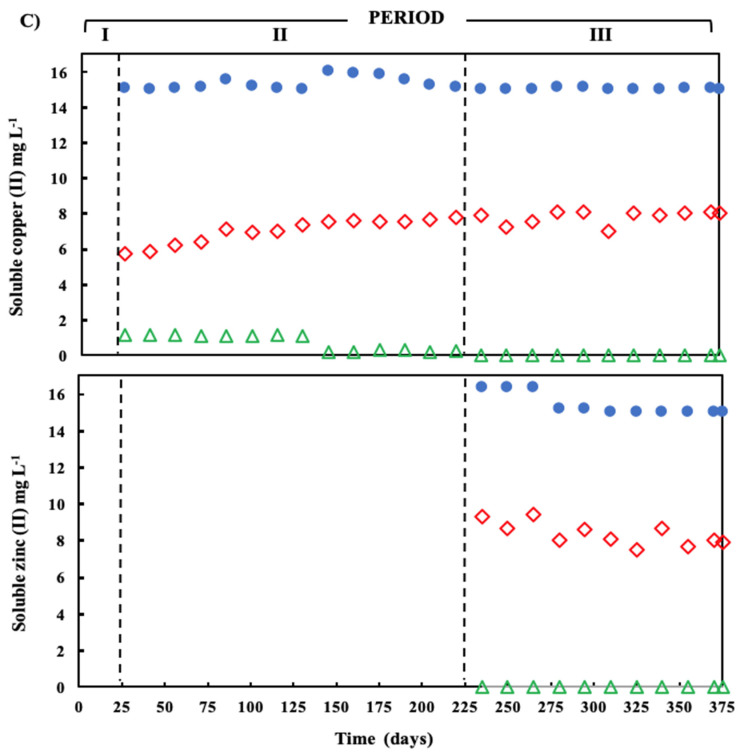
(**A**) pH variation in the operation time of the treatment system fed with a pH-2.7 synthetic ARD containing sulfate (2000 mg L^−1^), acetate as electron donor (2.5 g COD L^−1^), Cu(II) (15 mg L^−1^ during periods II and III) and Zn(II) (15 mg L^−1^ during period III): limestone pre-column influent (●), limestone pre-column effluent/bioreactor influent (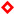), and bioreactor effluent (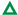). (**B**) Sulfate reduction (primary axis-left) and sulfide production (secondary axis-right) in the treatment system fed with a synthetic ARD containing sulfate (2000 mg L^−1^), acetate as electron donor (2.5 g COD L^−1^), Cu(II) (15 mg L^−1^ during periods II and III) and Zn(II) (15 mg L^−1^ during period III): sulfate (●) and sulfide (▲) in the influent and sulfate (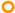) and sulfide (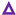) in the effluent. (**C**) Concentration of soluble Cu(II) and soluble Zn(II) during the operation of the treatment system fed with a synthetic ARD containing sulfate (2000 mg L^−1^), acetate as electron donor (2.5 g COD L^−1^), Cu (II) (15 mg L^−1^ during periods II and III) and Zn(II) (15 mg L^−1^ during period III): limestone pre-column influent (●), limestone pre-column effluent/bioreactor influent (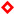), and bioreactor effluent (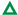).

Treatment of pH of the acidic ARD in the limestone pre-column during periods II and II increased the pH from 2.7 to neutral pH (Figure 2A). Following treatment in the SR bioreactor, the effluent pH values were 8.04, 7.45 and 7.43 in the periods I, II and III, respectively.

The average sulfate removal was approximately 38% in periods I and II (Table 1), reaching a sulfate removal of 48.8%. The average concentration of biogenic sulfide formed during the three operation periods was 160, 191 and 158 mg H_2_S-S L^−1^, respectively.

**Figure 3:**
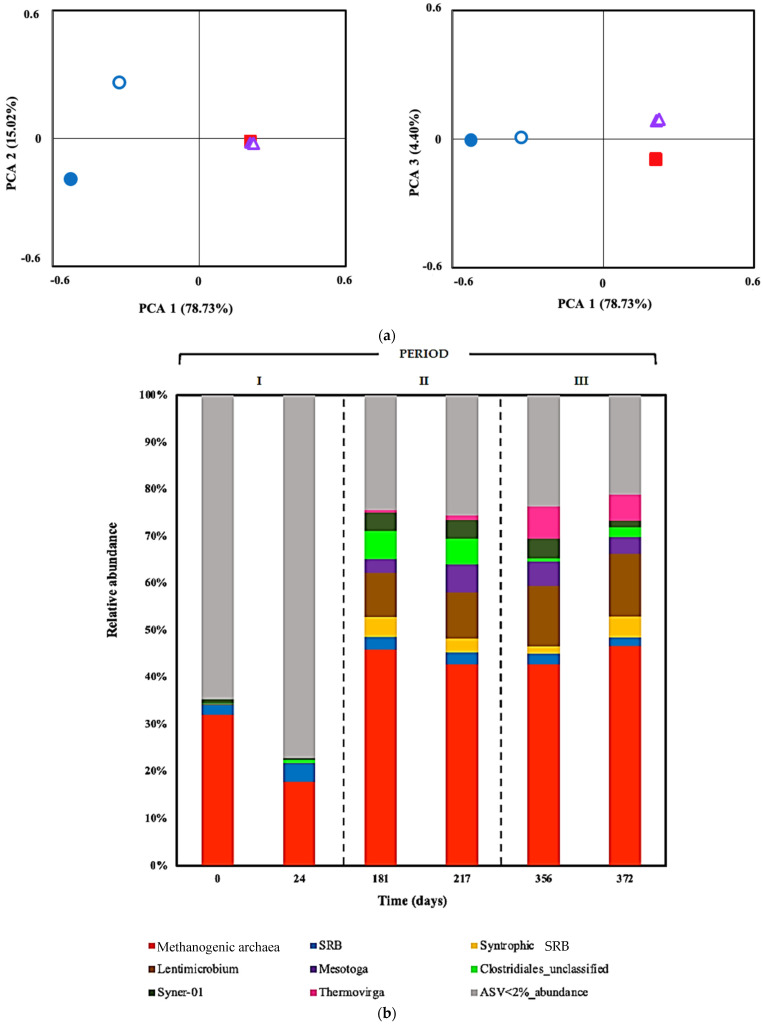
(**a**) Analysis performed on Bray–Curtis distances or dissimilarities for six sludge samples during the three different operation periods of the sulfate-reducing bioreactor with the limestone pre-column system. Two samples or biological replicates were collected in each period. Period I (adaptation phase): day 0 (●) and day 24 (**○**). Period II (15 mg Cu(II) L^−1^): day 181 (■) and day 217 (**□**). Period II (15 mg Cu(II) L^−1^ and 15 mg Zn(II) L^−1^: day 356 (▲) and day 372 (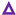). (**b**) Relative abundances of most abundant amplicon sequence variants (ASVs) at genus level in six sludge samples during the three different operation periods of the sulfate-reducing bioreactor with the limestone pre-column system. Two samples or biological replicates were collected in each period.

The organic COD removal efficiency increased by approximately 70% in relation to the initial COD concentration in the influent (Table 1). In the same manner, Table 1 summarizes the fraction of acetate (organic substrate), as %COD, used primarily by SRBs for H_2_S production; there was no methane production. The percentages of organic COD removed by SRB were 49.4, 51.3 and 42.4% during periods I, II and III, respectively. No significant statistical differences were observed. The time course of sulfate reduction showed reduction in sulfate concentration in all three periods, while sulfide concentration increased until the values stabilized (Figure 3B).

**Figure 4:**
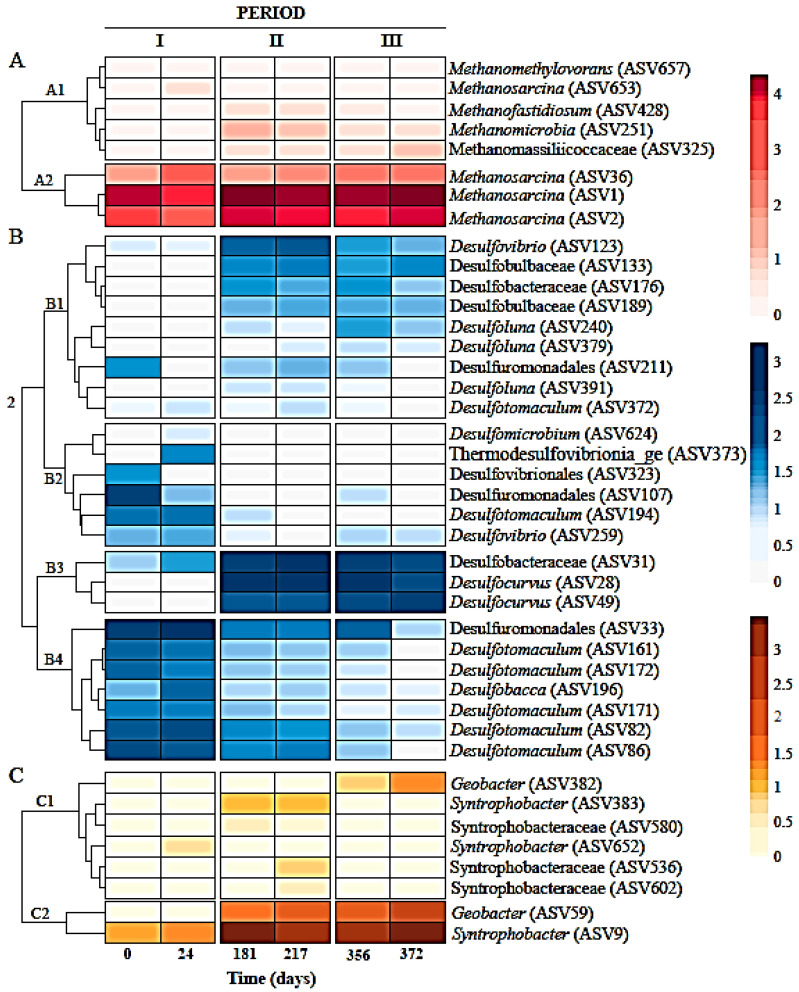
Clusters and heat maps plotting log_10_ of the counts for methanogenic archaea, SRB and syntrophic bacteria amplicon sequence variants (ASVs).

The sequential treatment system was highly effective for removing heavy metals. About a half of the initial Cu(II) and Zn(II) concentration was removed in the limestone pre-column and the remaining fraction in the bioreactor (Figure 4a). Cu(II) removal efficiency by the complete treatment system was 98.5 and 99.9%, in periods II and III, respectively, whereas Zn(II) removal during period III was close to 100%. In the limestone pre-column, copper removal efficiency was about 50% in periods II and III, and in period III Zn removal efficiency was 47.1% (Table 2). In the SR bioreactor, the average concentration of soluble Cu(II) was reduced from 6.98 to 0.22 mg L^−1^ in period II, and from 7.54 to 0.01 mg L^−1^ in period III. Zn(II) removal in the bioreactor was 51.8%.

### 3.2. Microbial Community Analysis

Genomic DNA from sludge samples collected in three operation periods of a sulfate-reducing bioreactor were used for 16S rRNA gene sequencing. A total of 69,000 reads per sample were obtained after the exclusion of singletons and rarefaction. All indices of alpha (α) diversity (Table 3) showed differences in reference to lesser values along the operation of the bioreactor. The Inverse Simpson Index, a measure of the richness in a community with a uniform evenness that would have the same level of diversity, decreased by approximately a half at period III compared to the adaptation stage.

Figure 3a shows beta (β) diversity statistics using a principal components plot (PCA) plotting the Bray–Curtis distance. These graphics show dissimilarities in the microbial composition of two sludge samples obtained during period I or adaptation phase compared with periods II and III, and that >93% of the variance was explained by changes in pH and the addition of metals to ARD fed to the treatment system. Conversely, there were no significant differences in microbial communities between samples of periods II and III, showing the same cluster patterns; the second PCA plot shows a better differentiation among assemblages at periods II and III explained by the addition of Zn(II) during period III.

To analyze community structure, the relative prokaryotic abundances were organized at genus level in six sludge samples obtained during the three different operation periods of the treatment system (Figure 3b). The archaeal community was represented by methanogens, with relative abundance ranging from 32.1% to 46.7% in the sludge samples. The non-syntrophic SRB population remained stable, around a relative abundance of 2.0–1.8%. SRB syntrophic bacteria became significantly more abundant, 0.02% to 4.4%. Members of the genus *Lentimicrobium* were present from 0.1% at the beginning, and achieving 13.4% of total encountered ASVs at the end of the study. In period I, microbial populations belonging to the genus *Mesotoga* presented a very low abundance proportion (0.1%) and increased their relative abundance to 3.0 to 6% in period II, but they decreased in period III by approximately ~3.5 to 1.5%. Unclassified *Clostidriales* had low abundance (0.1%) in sludge samples during period I or the adaptation phase and increased their relative abundance to 3.0 to 6% in period II, but they decreased in period III by 0.7 to 2.0%. ASVs corresponding to *Syner-01* increased from a relative abundance of 1% at the beginning of the period I to around 4% in period II and finally decreased to 1.5%. *Thermovirga* was not detected during period I and then increased to 5.6%. Other ASVs with relative abundance less than 2% reduced from 64.6 to 21.1% in the time course of the bioreactor operation with the limestone pre-column system.

Figure 4 shows heatmaps clustered as methanogenic archaea, non-syntrophic and syntrophic sulfate-reducing bacteria ASVs. Methanogens included the genera *Methanosarcina* (ASV1, ASV2, ASV36 and ASV653), *Methanomicrobia* unclassified (ASV251), *Candidatus Methanofastidiosum* (ASV428), *Methanomethylovorans* (ASV657), and *Methanomassiliicoccaceae* (ASV325). The most abundant methanogens in all sludge samples were *Methanosarcina* and those populations increased with bioreactor operation, except ASV653 that was relatively stable, as well as the remainder of the identified methanogens. Sulfate-reducing bacteria were represented by members of *Desulfocurvus* (ASV28 and ASV49), Desulfobacteraceae (ASV31 and ASV176), *Desulfuromonadales* (ASV33, ASV107 and ASV211), *Desulfotomaculum* (ASV82, ASV86, ASV161, ASV171, ASV172, ASV194 and ASV372), *Desulfovibrio* (ASV123 and ASV259), *Desulfobulbaceae* (ASV133 and ASV189), *Desulfobacca* (ASV196), *Desulfoluna* (ASV240, ASV379 and ASV391), *Desulfovibrionales* (ASV323), *Thermodesulfovibrionia*_ge (ASV373) and *Desulfomicrobium* (ASV624). Sulfate-reducing populations belonging to genera *Desulfobacteraceae*, *Desulfocurvus*, *Desulfobulbaceae* and *Desulfovibrio* became more abundant, while *Desulfuromonadales*, *Desulfotomaculum* and *Desulfobacca* decreased. Syntrophic SRB corresponded to genera *Geobacter* (ASV59 and ASV3829), *Syntrophobacter* (ASV9, ASV383 and ASV652), and members of *Syntrophobacteraceae* (ASV536, ASV580 and ASV602). *Syntrophobacter* (ASV9) and ASVs belonging to the *Geobacter* genus were enriched with bioreactor operation. Other ASVs affiliated to SRB syntrophic bacteria remained stable.

**Figure 5:**
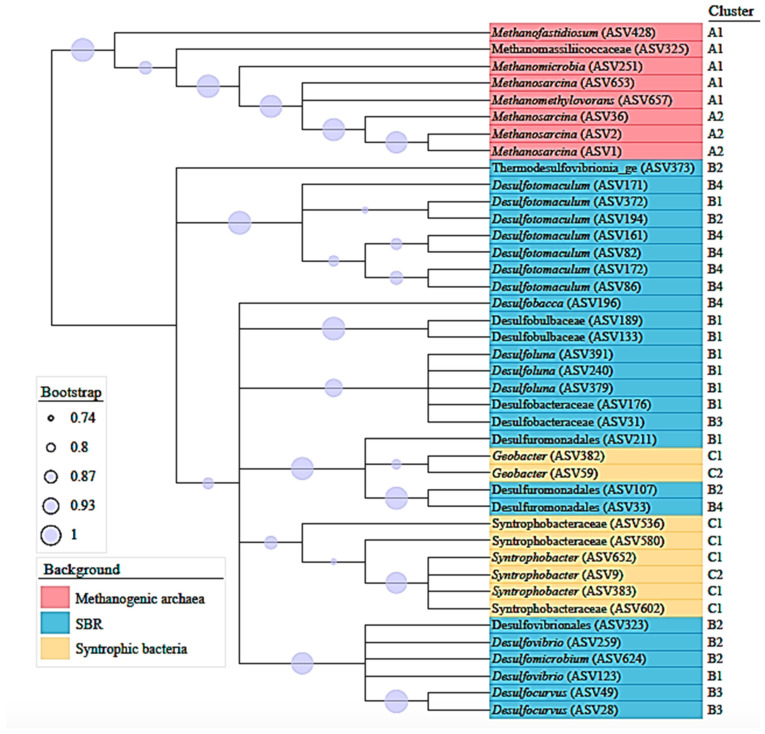
Phylogenetic tree based on V3 and V4 regions of 16SrRNA sequences datasets, showing communities of methanogenic archaea (red), SRB (blue) and syntrophic bacteria (yellow). The branches correspond to amplicon sequence variants (ASVs) reported in this study. Bootstrap values are shown as circles.

Forty-one ASVs were considered to build a consensus tree using the V3 and V4 regions of 16SrRNA sequences datasets (Figure 5). A phylogenetic tree that represented methanogenic, non-syntrophic and syntrophic sulfate reducing populations, that grouped 8, 25 and 8 ASVs, respectively, was constructed. In addition, the analysis included reference nomenclature of the clusters obtained from heatmaps represented in Figure 4. *Methanosarcina* ASVs belonging to cluster A2, the most abundant methanogens in this study, were as closely related to *Methanosarcina* (ASV 653) as *Methanomethylovorans*, both with low abundance from cluster A1. Therefore, according to the consensus tree, the bacterial community was represented as phylogenetically coherent groups, including sequences of non-syntrophic SRBs and syntrophic SRBs.

## 4. Discussion

### 4.1. Bioreactor Performance: Sulfate Removal, Sulfide Production and Metals Removal

Sulfate removal is highly related to the ability of the treatment system to promote bioprecipitation of metals [52]. In the same bioreactor, Mendez et al. reported continuous increase in sulfate removal, reaching a maximum value of 35.7% when synthetic ARD was supplemented with Cu(II) concentrations of 10, 20, 30 and 40 mg L^−1^ [30]. While, our results showed better sulfate reducing activity, suggesting that growing of SRBs and sulfidogenesis were not affected by the presence of Cu(II) and Zn(II). This could be due to a better adaptation by SRBs in sludge, or to the combination of two or more metals in higher concentrations [53,54,55,56].

Previous research evaluated the inhibitory concentrations of Cu(II) and Zn(II), alone or in a mixture, using sludge inoculated in an SR bioreactor with a limestone pre-column system [57]. Those results showed that 15 mg L^−1^ of Cu(II) caused approximately 30% inhibition of the sulfate reducing activity. On the other hand, 15 mg Cu(II) L^−1^ and 15 mg Zn(II) L^−1^ caused less than 20% inhibition. In addition, depending on the nature of the metals in the mixture, their influence over anaerobic digestion can generate synergistic or antagonistic effects [19,52]. Despite this, no inhibitory effect due to Cu(II) and/or Zn(II) presence was observed during periods II and III of operation of the SR bioreactor in the current study. This could be attributed, mainly, to the low concentrations of these metals in the bioreactor, since in the limestone pre-column about 50% metal precipitation occurs as hydroxides and carbonates [53]. Moreover, the chemisorption in the sand (solid phase) into the bioreactor, could provide support and protection to anaerobes of the inoculated sludge through formation of biofilm [58].

The absence of methane in the SR bioreactor is not surprising since SRB can compete better than methanogens for common organic and inorganic substrates [52]. Typically, the organic substrate affinity of SRB for acetate is ten-fold higher than that of methanogens; consequently, the result of this competition is methanogenic inhibition, and methanogenesis is the predominant process [5,14,15]. On the other hand, an additional inhibition effect could be attributed to the toxicity of sulfide, the product of sulfate reduction, for other microbial groups [52]. For example, Moosa and Harrison [59] reported microbial inhibition in terms of a reduction in sulfate reduction activity and an increase when sulfide concentration increased in a chemo-stat culture at pH 7.0 ± 0.2 during the treatment of acid mine drainage. In addition, an excess of sulfate (e.g., 0.6 g COD/g SO_4_^2−^), could promote the dominance of SRBs over methanogens at pH levels ranging from 7.4 to 8.0 [20]. Moreover, the presence of Cu(II) and/or Zn(II) can be toxic to methanogens and other microorganisms, through mechanisms such as the generation of reactive species of oxygen, or transmembrane interference in nutrient and energy transport, among others; consequently, methanogenesis could be affected [53,54,60]. Toxicity threshold concentrations of Cu(II) and Zn(II) to methanogens have been reported in several studies in the literature, which vary widely depending on the conditions under which anaerobic degradation was evaluated [61,62,63].

The complete treatment system composed of the limestone pre-column and the bioreactor efficiently removed Cu(II) and Zn(II). The limestone pre-column drove ARD neutralization and promoted metal precipitation as carbonates and hydroxides [53,64]. On the other hand, the bioreactor demonstrated strong Cu(II) and Zn(II) removal efficiencies through bioprecipitation. Sierra-Alvarez et al. [20] reported Cu(II) removal efficiencies higher than 99% in a system integrating a SR bioreactor with a fluidized bed crystallization reactor for semiconductor manufacturing wastewater treatment. In a study evaluating the bioremediation of ARD in flow-through columns testing zero-valent iron (ZVI), Ayala-Parra et al. obtained high removal efficiencies of Cu(II), Cd(II) and Pb(II) [53]. In bioreactors for treatment of ARD with very high concentrations of metals, efficiencies of the order of 90% and higher were achieved during the bioprecipitation of Cu (II), Zn (II), Cd (II), Pb (II), Ag (II), and Fe (II) [65].

Our data indicate that biogenic sulfide production was a confirmation of Cu(II) and Zn(II) removal by bioprecipitation. Nevertheless, pH neutralization and abiotic metal precipitation that occurred in the limestone pre-column, significantly contributed to heavy metal immobilization [30]. For that reason, the limestone in the bed reactor should be replaced when exhausted [66].

### 4.2. Microbial Diversity and Community Structure

Alpha diversity refers to which habitat contains the most taxa within each sample, while beta diversity refers to the habitat within samples [67,68,69]. This study showed a reduction in alpha and beta diversity with bioreactor operation time. This was supported by concomitant reduction of the richness, evenness, and dissimilarity metrics [69,70,71,72]. In addition, our findings indicated a higher dynamic alteration of low abundance ASVs and a stable persistence of a few dominant ASVs that contributed to the sulfate reduction process.

In bioreactors treating ARD, methanogens, such as *Methanobacterium*, *Methanosaeta* and *Methanosarcina*, among others, have been detected [14,30,73]. In the present research, the archaeal community was mainly constituted by ASVs belonging to *Methanosarcina*, which is the most abundant group of ASVs in the prokaryotes of all sludge samples assessed. These archaea are mainly acetoclastic methanogens, a robust and tolerant group against different stressors compared to other methanogens [14,74]. However, the *Methanosarcina* genus also includes hydrogenotrophic and methylotrophic methanogens [75]. *Methanosarcina* dominated the archaeal community, possibly displacing other methanogens. De Vrieze et al. [74] reported that the growth rate of *Methanosarcina* spp. is higher than those of *Methanosaeta* species in mesophilic anaerobic digestion with acetate concentrations up to 100 mg L^−1^. Differences in growth rates could generate the dominance of *Methanosarcina* populations over other methanogen species in the archaeal community.

Even though methanogens were the most abundant microorganisms, no methane production was detected. In addition to inhibition due to the sulfate reduction process, the absence of methane could be hypothesized to occur in the following two possible scenarios: (1) biological oxidation of methane or methanotrophy, that could occur by a combination of biotic and abiotic factors. Methanotrophy is driven by bacteria affiliated with *Gamma-proteobacteria* and *Alpha-proteobacteria* classes and anaerobic methanotrophic archaea (ANME) [76]. (2) reverse methanogenesis—this chemical mechanism occurs when SRBs consume hydrogen entirely, then methane concentration increases and the reverse reaction is thermodynamically possible [76,77]. In addition, due to the versatile and complex metabolisms of members of *Methanosarcina*, literature studies report that they are capable of growing and competing under anaerobic conditions without producing methane [78]. Finally, methane analysis is susceptible to being optimized throughout the employment of gas chromatography for detection and quantification [79].

In the current investigation, all non-syntrophic SRB populations increased during bioreactor operation and were affiliated to the *Deltaproteobacteria* class. While those which decreased (i.e., *Desulfuromonadales*, *Desulfotomaculum* and *Desulfobacca*), were affiliated to the Deltaproteobacteria and Clostridia classes [5]. The class *Deltaproteobacteria* is recognized to be a monophyletic bacteria group which are Gram-negative staining, mesophilic, sulfur and sulfate reducing, and able to reduce elemental metals [80]. On the other hand, in the phylum *Firmicutes*, *Clostridia* is a polyphyletic class that groups mainly Gram-positive bacteria and includes species of the *Desulfotomaculum* genus [5,81]. According to the results, the metal concentrations tested in the current study did not cause upset by changes in function and structure of enzymes that intervene in the sulfate reducing process [30]. This is due to the presence of metals that could generate endogenous dynamics in the composition of SBRs and select resistant populations. Mendez-Garcia et al. [25] reported that the presence of these microorganisms at acid mine drainage habitats is restricted to *Deltaproteobacteria* and *Firmicutes* members. A deeper biological knowledge of microbial resistance strategies to heavy metals requires an evolutionary genetics, proteomics and metabolomics approach [82,83].

Species of *Geobacter* and *Syntrophobacter* are acetate-oxidizing bacteria, and capable of metal reduction and sulfate reduction [25,46]. Both are affiliated with the *Deltaproteobacteria* class and became markedly more abundant during operation of the SR bioreactor [5,81]. A potential syntrophic partnership between bacteria, including *Geobacter* and *Syntrophobacter*, and hydrogenotrophic methanogens has been reported when acetoclastic methanogens, such as *Metanosarcina* spp., become more abundant in a sulfidogenic dominant environment [15,16,84].

As expected, the consensus tree provides a robust phylogenetic framework for the current study. At class level, we obtained clusters following the taxonomy, traits, and dynamics of microorganisms during the bioreactor operation [29,85].

Further studies are needed to determine how some factors, such as utilization of sustainable sources of electron donors (e.g., lignocellulosic or non-lignocellulosic materials) may play a significant role in microbiological interactions during sulfate reducing processes. In addition, it is relevant to study other microbial populations with syntrophic and fermentative lifestyles, and their metabolic networks.

## 5. Conclusions

This study confirmed that the treatment system, integrating a limestone pre-column and an SR bioreactor, is a successful technology for the removal of Cu(II) and Zn(II) from synthetic ARD using acetate as an organic carbon source and sulfate. Both metals were removed in the limestone pre-column and in the sulfate-reducing bioreactor.

The microbial community was comprised of archaeal and bacterial populations. However, sulfidogenesis was the dominant process and methanogenesis was not observed. An analysis of microbial diversity demonstrated changes throughout stabilization of the sulfate-reducing process and before the addition of metals. Gradually, the microbial community in the bioreactor became less diverse and interactions resulted in syntrophic associations. Thereby, the bioprecipitation process was not affected.

These findings provide new insights for understanding the metabolism and the functionality of microbial populations involved in sulfate reducing processes.

## Figures and Tables

**Table 1:** Set up of the experiment, influent composition, and conditions of operation of the treatment system during the three periods of operation.

Period ^a^	Time of Operation ^b^ (d)	Influent	Effluent pH	Sulfate Removal (%)	Effluent Sulfide (mg S L^−1^)	%CODin ^c^		
		pH				H_2_S Formed	CH_4_	Organic COD Removal
I ^d^	24	8.04 ± 0.36	8.04 ± 0.36	37.7 ± 4.8	151.0 ± 20.1	49.4 ± 7.1	0.0 ± 0.0	68.1 ± 7.48
II	196	7.45 ± 0.15	7.45 ± 0.15	38.0 ± 12.3	179.8 ± 19.6	51.3 ± 12.6	0.0 ± 0.0	74.0 ± 5.09
III	155	7.43 ± 0.21	7.43 ± 0.21	48.8 ± 5.1	149.1 ± 18.0	42.4 ± 10.1	0.0 ± 0.0	73.0 ± 4.82

^a^ The average acetate and sulfate concentrations in the influent were 2500 mg COD L^−1^ and 670 mg L^−1^, respectively, during all periods of operation. ^b^ Hydraulic retention time (HRT) in the bioreactor was approx. 1 day and temperature was 30 ± 2 °C. ^c^ Values are expressed as percentage of the initial wastewater COD (COD_in_). ^d^ Adaptation period, stand-alone sulfate-reducing bioreactor.

**Table 2:** Average removal of soluble Cu(II) and Zn(II) attained by the treatment system during the various periods of operation.

Period ^a^	Metal in the Influent		Removal of Soluble Metal (%)					
			Limestone Pre-Column		Bioreactor		Complete System	
	(mg Cu(II) L^−1^)	(mg Zn(II) L^−1^)	Cu(II)	Zn(II)	Cu(II)	Zn(II)	Cu(II)	Zn(II)
II	15.33 ± 0.37	-	54.5 ± 0.8	-	96.8 ± 0.8	-	98.5 ± 0.6	-
III	15.17 ± 0.35	15.54 ± 0.60	50.3 ± 1.9	47.1 ± 0.7	99.8 ± 0.9	99.9 ± 1.0	99.2 ± 0.4	>99.9 ± 1.0

^a^ Heavy metals were not present in the influent during period I (adaptation).

**Table 3:** Alpha diversity indices of the mixed microbial culture during the various periods of operation of the sulfate-reducing bioreactor.

Period	Time (d)	Shannon	Inverse Simpson	Richness	Chao1	Evenness
I	0	3.64	13.24	306.00	306.86	0.64
	24	3.90	23.11	250.00	250.00	0.71
II	181	2.88	7.28	272.00	278.11	0.51
	217	2.99	8.07	277.00	278.56	0.53
III	356	2.93	7.72	246.00	246.43	0.53
	372	2.73	6.77	217.00	219.57	0.51

## Data Availability

Amplicon sequencing data, count tables and relevant data files can be found at GitHub (https://github.com/darioxr/SR_bioreactor_Cu_Zn (accessed on 8 January 2022)). This research was carried out with permit number MAE-DNB-CM-2018-0085 of the Ecuadorian Ministry of the Environment.
